# Chronic kidney disease progression: a retrospective analysis of 3-year adherence to a low protein diet

**DOI:** 10.1080/0886022X.2017.1282374

**Published:** 2017-02-02

**Authors:** Felipe Rizzetto, Viviane de Oliveira Leal, Leonardo Soares Bastos, Denis Fouque, Denise Mafra

**Affiliations:** a Hospital Federal da Lagoa, Rio de Janeiro, Brazil;; b Division of Nutrition, Pedro Ernesto University Hospital, State University of Rio de Janeiro (UERJ), Rio de Janeiro, Brazil;; c Scientific Computing Program, Oswaldo Cruz Foundation (FIOCRUZ), Rio de Janeiro, Brazil;; d Department of Nephrology, Centre Hopitalier Lyon Sud, INSERM 1060, CENS, Université de Lyon, Pierre-Benite, France;; e Graduate Program in Medical Sciences, Federal Fluminense University (UFF), Niterói, Rio de Janeiro, Brazil

**Keywords:** Chronic renal insufficiency, adherence, protein-restricted diet, glomerular filtration rate

## Abstract

The potential benefits and dangers of dietary protein restriction in chronic kidney disease (CKD) are still controversial. Thus, the aim of this study is to evaluate the effect of low protein diet (LPD) on the renal function in nondialysis CKD patients. A retrospective study was conducted from 321 nondialysis CKD patient’s medical files (65.1 ± 12.7 yrs, 58.2% men). These patients received individualized dietary protein prescription (0.6–0.8 g protein/kg/day). Protein intake was evaluated by food diary and 24 h-food recall. Adherence to the LPD was considered when patients intake from 90 to 110% of the prescribed amount of protein. The patients were divided into 4 groups: (G1) adherent diabetes mellitus (DM) patients (*n* = 83); (G2) non-adherent DM patients (*n* = 106); (G3) adherent non-DM patients (*n* = 75); (G4) non-adherent non-DM patients (*n* = 57). Renal function was assessed by estimated glomerular filtration rate (eGFR). Both groups of patients (DM and non-DM) that adhered to the LPD showed significant improvement in eGFR (G1: 38.7 ± 13.2 mL/min to 51.1 ± 17.0 mL/min (*p* < 0.001); G3: 35.1 ± 16.8 mL/min to 46.8 ± 21.4 mL/min (*p* < 0.001)). In adherent patients, no differences in albumin and BMI were observed at the end of follow up. In non-adherent patients, eGFR significantly decreased in DM group (G2: 44.2 ± 18.5 mL/min to 38.2 ± 15.8 mL/min (*p* = 0.003)). According to multivariate analysis, annual changes in eGFR were not independent associated with age, gender, BMI, lipid profile, bicarbonate or smoking status. In summary, adherence to low protein diet could be able to improve serum creatinine and eGFR, well-known markers of renal function. However, prospective studies are needed to control confounders which affect renal function and CKD progression.

## Introduction

For healthy adults, the Recommended Dietary Allowance (RDA) for protein is 0.8 g/kg/day. This amount represents dietary protein requirements (0.6 g/kg/day) plus a percentage to achieve the safe intake^1^ and it is quite similar to the recommendation for protein intake of nondialysis chronic kidney disease (CKD) patients proposed by the National Kidney Foundation (0.6 to 0.8 g protein/kg/day).^2^ However, the term “low protein diet” (LPD) is used to describe this recommendation because a diet restricted to 0.6 g protein/kg/day is 25% below the recommended (0.8 g protein/kg/day).^3^

The benefits of dietary protein restriction for nondialysis CKD patients include reduction of hyperphosphatemia, metabolic acidosis, hyperkalemia and uremic toxins that may suppress the appetite and stimulate muscle protein wasting.^2^^,^^4–6^ However, the role of dietary protein restriction in slowing CKD progression is still controversial.^4^

Primary results of Modification of Diet in Renal Disease (MDRD), the largest randomized clinical trial in this matter, were not conclusive with regard to the effectiveness of the LPD on CKD progression.^7^ However, secondary analysis suggested many beneficial effects of protein restriction.^8^ Thus, studies on the effects of LPD in the CKD progression were performed and generated non-conclusive or biased studies in part due to the diet adherence,^9–11^ which is key to success.^12^^,^^13^

Although well-conducted randomized controlled trials are best for studying short-term efficacy, observational studies may be more appropriate when the intervention depends on the patient’s active participation.^14^ In practice, the long-term effects of LPD should be evaluated face the adherence to the diet.^12^ Poor adherence has been described^8^^,^^11^ and, consequently, strategies to improve the LPD adherence, as intensive nutritional counseling are highly recommended.^2^ Thus, the aim of this study was to evaluate the long-term effects of protein restriction on renal function according the LPD adherence.

## Methods

A retrospective analysis of 480 patients’ files attending the conservative treatment from renal nutrition ambulatory of the Hospital da Lagoa (Rio de Janeiro, Brazil) between June 2008 and January 2013 was performed. The patients (without previous nutritional counseling) were instructed to modify their intake of proteins, sodium and phosphorus and, if necessary, caloric intake in order to achieve the goals of the assigned diet. Dietary instructions and the verification of adherence to the prescribed diet were accomplished by a single expert renal dietitian that followed all patients included in the study at each CKD-clinical visit (every 3 months).

The inclusion criteria were age >18 years and estimated glomerular filtration rate (eGFR) < 60 mL/min. The average follow-up was 3 years (≅4 clinic visits per year) and patients who were followed for less than 3 years, less than 4 visits per year and those who had their treatment interrupted by absence during this period were excluded. Moreover, patients with protein intake less than 90% of dietary prescription were also excluded. A total of 321 patients (65.1 ± 12.7 years of age, 58.2% men) without renal disease etiology selection entered the study. The study protocol was approved by the Ethics Committee of Medicine Faculty of Federal University Fluminense (n. 144/11).

All participants received dietary counseling as recommended by the Kidney Disease Outcomes Quality Initiative nutritional guidelines (0.6–0.8 g/kg/day of protein and 30–35 kcal/kg/day of energy) [2]. The adherence to dietary prescription was assessed by the 2 food diary (1 weekday and 1 weekend, nonconsecutive), where it was requested that the patient records all food and beverages consumed, its specificities (e.g., skim milk) and portions (in household measures, e.g., 1 cup of 200 mL). During nutritional consultations, the patients completed a 24-h food recall. In this case, patients were carefully instructed by the dietitian to record all kinds and amounts of food (including beverages) ingested, using measuring tools to estimate portion sizes and to improve the accuracy of record. Analyzes of these three food records were conducted with software developed by the Universidade Federal de São Paulo – Nutwin^®^. The nutrient contents of foods not contained in this software were searched on Brazilian Table of Food Composition.^15^

Good adherence to LPD was defined as medium protein intake between 90 and 110% of dietary prescription. To evaluate the understanding and motivation to the diet, it was realized a self-report question which the adherence was categorized as “excellent”, “very good”, “fair” and “poor”. At each visit a complete clinical and dietetic evaluation were performed and treatments were adjusted. Thus, the 321 enrolled patients were divided into 4 groups: Group 1: Diabetes mellitus (DM) patients who adhered to the diet (*n* = 83); Group 2: DM patients who did not adhered to the diet (*n* = 106); Group 3: Non-DM patients who adhered to the diet (*n* = 75); Group 4: Non-DM patients who did not adhered to the diet (*n* = 57). Serum creatinine, urea, albumin, lipid profile, and venous bicarbonate levels were evaluated at baseline and every 3 months. For DM patients, glucose and glycated hemoglobin (Hb) were also determined at each 3 months. Serum albumin was determined by bromocresol green method (normal range: 3.5–4.8 g/dL). Body mass index (BMI) was calculated according to the formula: weight/(height2). Renal function was expressed as eGFR, obtained by Chronic Kidney Disease Epidemiology Collaboration (CKD-EPI) creatinine equation,^16^ and the change of eGFR per year was also calculated. Current smoking status was investigated in Hospital database.

The Kolgomorov–Sminorv normality test was used to characterize the data distribution. Results are presented as mean ± standard deviation, median (interquartile range) or percentage, as applicable. Comparisons between baseline and the follow-up data were analyzed using paired *t*-test. For comparisons between independent groups, Student's *t* test, Chi-square or ANOVA test were used. Multivariate regression analysis was performed to verify variables independent associated with annual changes in eGFR. Statistical significance was accepted as *p* values <0.05.

## Results

The percentage of patients who adhered to LPD diet was 49.2% (25.8% DM and 23.4% non-DM). Age, gender, and BMI were not different among 4 groups. At baseline, creatinine levels and eGFR were not different between G1 and G2 and also between G3 and G4. Bicarbonate levels were lower in diabetic patients when compared with non-diabetic patients (Table 1). The hypertensive nephrosclerosis was the main etiology of CKD in non-DM patients (87% and 90% in group 3 and 4, respectively). The group 4 presented higher prevalence of current smoking status (19%, *p* < 0.05) when compared with patients on G1 (4.8%), G2 (6.9%), and G3 (8.3%).

**Table 1.** Baseline characteristics of CKD patients groups.

	Group 1	Group 2	Group 3	Group 4
Sample size (*n*)	83	106	75	57
Age (years)	68.0 ± 11.2	63.2 ± 11.6	66.5 ± 13.4	63.0 ± 14.8
Gender (% men)	57	58.5	60	64
Creatinine (mg/dL)	1.91 ± 0.61^a^^,^^b^	1.90 ± 0.70^c^^,^^d^	2.20 ± 0.84	2.23 ± 0.81
eGFR (mL/min)	38.7 ± 13.2^a^^,^^b^	44.2 ± 18.5^c^^,^^d^	35.1 ± 16.8	35.1 ± 18.2
BMI (kg/m^2^)	25.8 ± 5.5	28.2 ± 5.6	27.2 ± 5.1	27.6 ± 5.8
Cholesterol (mg/dL)	187.8 ± 48.7	187.2 ± 51.5	192.7 ± 54.3	195.5 ± 47.9
Triglycerides (mg/dL)	158.9 ± 65.3	163.0 ± 94.9	151.8 ± 99.5^e^	221.5 ± 99.9
HDL (mg/dL)	42.0 ± 10.3	42.0 ± 9.6	45.3 ± 29.1	43.3 ± 13.6
Glycated Hb (%)	6.3 ± 1.1^f^	7.4 ± 1.4	–	–
Bicarbonate (mM)	21.1 ± 2.4^a^^,^^b^	21.3 ± 1.7^c^^,^^d^	22.7 ± 2.3	22.3 ± 2.0

a *p* < 0.05 between G1 and G3.

b *p* < 0.05 between G1 and G4.

c *p* < 0.05 between G2 and G3.

d *p* < 0.05 between G2 and G4.

e *p* < 0.05 between G3 and G4.

f *p* < 0.05 between G1 and G2.

BMI: body mass index; eGFR: estimated glomerular filtration rate; HDL: high density lipoprotein; Hb: hemoglobin. Group 1: DM (diabetes mellitus) patients who adhered to diet; Group 2: DM patients who not adhered to diet; Group 3: Non-DM patients who adhered to diet; Group 4: Non-DM patients who not adhered to diet.

The clinical and biochemical characteristics of studied CKD patients are presented in Table 2. In adherent patients (DM and non-DM), creatinine levels decreased significantly after nutritional intervention when compared with patients who did not adhered to LPD. It was also observed increase in the eGFR after follow-up nutritional counseling for patients who adhered to the diet and the serum albumin levels remained within normal values (>3.8 g/dL) in these patients. In addition, there was no change in body weight in these groups. Although non-significant, the albumin levels were reduced and BMI increased in non-adherent patients (G2 and G4) after follow-up (it was not observed fluid overload in these patients).

**Table 2.** Anthropometric and biochemical data at the beginning and at end of the follow-up period.

Groups	Before	After	*p* Values
Group 1 (*n* = 83)			
Creatinine (mg/dL)	1.91 ± 0.61	1.63 ± 0.72	0.002
eGFR (mL/min)	38.7 ± 13.2	51.1 ± 17.0	<0.001
Urea (mg/dL)	64.2 ± 13.2	60.9 ± 17.0	0.105
Albumin (g/dL)	4.1 ± 0.3	4.2 ± 0.2	0.413
BMI (kg/m^2^)	25.8 ± 4.4	25.9 ± 4.6	0.579
Cholesterol (mg/dL)	187.8 ± 48.7	183.1 ± 48.2	0.599
Triglycerides (mg/dL)	158.9 ± 65.3	155.6 ± 48.4	0.793
HDL (mg/dL)	42.0 ± 10.3	45.1 ± 11.1	0.183
Fasting glucose (mg/dL)	140.9 ± 69.9	113.1 ± 26.9	0.03
Glycated Hb (%)	6.3 ± 1.1	6.2 ± 1.0	0.527
Bicarbonate (mM)	21.1 ± 2.4	22.4 ± 1.4	<0.001
Group 2 (*n* = 106)			
Creatinine (mg/dL)	1.90 ± 0.70	2.03 ± 0.71	0.153
eGFR (mL/min)	44.2 ± 18.5	38.2 ± 15.8	0.003
Urea (mg/dL)	76.6 ± 38.3	78.5 ± 31.4	0.302
Albumin (g/dL)	4.0 ± 0.3	3.7 ± 0.1	0.142
BMI (kg/m^2^)	28.2 ± 5.6	28.7 ± 5.9	0.489
Cholesterol (mg/dL)	187.2 ± 51.5	196.4 ± 50.1	0.200
Triglycerides (mg/dL)	163.0 ± 94.9	189.0 ± 99.9	0.260
HDL (mg/dL)	42.0 ± 9.6	40.6 ± 6.8	0.417
Fasting glucose (mg/dL)	142.0 ± 66.5	163.1 ± 61.8	0.089
Glycated Hb (%)	7.4 ± 1.4	7.2 ± 1.3	0.408
Bicarbonate (mM)	21.3 ± 1.7	23.1 ± 1.6	<0.001
Group 3 (*n* = 75)			
Creatinine (mg/dL)	2.20 ± 0.84	1.82 ± 0.82	0.03
eGFR (mL/min)	35.1 ± 16.8	46.8 ± 21.4	<0.001
Urea (mg/dL)	70.9 ± 25.2	62.6 ± 26.3	0.05
Albumin (g/dL)	4.1 ± 0.4	3.9 ± 0.3	0.122
BMI (kg/m^2^)	27.5 ± 5.1	27.7 ± 5.5	0.545
Cholesterol (mg/dL)	192.7 ± 54.3	194.0 ± 38.6	0.680
Triglycerides (mg/dL)	151.8 ± 99.5	122.8 ± 61.0	0.200
HDL (mg/dL)	45.3 ± 29.1	46.3 ± 29.3	0.600
Bicarbonate (mM)	22.7 ± 2.3	22.6 ± 3.2	0.871
Group 4 (*n* = 57)			
Creatinine (mg/dL)	2.23 ± 0.81	2.42 ± 1.04	0.136
eGFR (mL/min)	35.1 ± 18.2	33.7 ± 16.9	0.178
Urea (mg/dL)	70.9 ± 31.2	71.7 ± 31.5	0.267
Albumin (g/dL)	3.9 ± 0.4	3.2 ± 0.3	0.090
BMI (kg/m^2^)	27.6 ± 5.8	29.8 ± 6.0	0.101
Cholesterol (mg/dL)	195.5 ± 47.9	191.5 ± 29.6	0.367
Triglycerides (mg/dL)	221.5 ± 99.9	205.1 ± 63.0	0.657
HDL (mg/dL)	43.3 ± 13.6	41.8 ± 9.1	0.157
Bicarbonate (mM)	22.3 ± 2.0	23.3 ± 1.7	0.045

BMI: body mass index; eGFR: estimated glomerular filtration rate; HDL: high density lipoprotein; Hb: hemoglobin. Group 1: DM patients who adhered to diet; Group 2: DM patients who not adhered to diet; Group 3: Non-DM patients who adhered to diet; Group 4: Non-DM patients who not adhered to diet.

Except in adherent non-diabetic patients (G3), venous bicarbonate showed significant increase in follow-up time. Bicarbonate levels were not related to annual changes in eGFR. The change of eGFR by year was statistically different between adherent and non-adherent patients (G1: 3.6 (5.5) mL/min/year; G2: −1.67 (5.8) mL/min/year; G3: 4.4 (7.7) mL/min/year; G4: −1.33 (8.1) mL/min/year; *p* < 0.0001 between groups G1 and G2; G3 and G4). It was not observed statistical difference in change of eGFR between G1 and G3; G2 and G4. Multivariate regression analysis were performed to evaluate the independent influence of variables possibly associated with progression of CKD (age, gender, smoking status, BMI, cholesterol, triglycerides, high density lipoprotein (HDL) and bicarbonate) on annual changes of eGFR (Table 3). None of these variables was independent associated with change of eGFR by year.

**Table 3.** Regression analysis for the independent determinants of annual changes in estimated glomerular filtration rate.

Variable	Β coefficient	Standard error	*p* Values
Age	0.082	0.065	0.570
Gender	−0.106	1.885	0.515
Smoking	−0.081	3.056	0.572
Body mass index	0.171	0.176	0.277
Cholesterol	0.139	0.021	0.398
Triglycerides	−0.146	0.009	0.369
High density lipoprotein	−0.155	0.078	0.376
Glycated hemoglobin	−0.125	0.667	0.411
Bicarbonate	−0.044	0.379	0.772

## Discussion

We investigated the role of LPD adherence on renal function during 3 years of intensive and specialized nutritional counseling. The patients who adhered to the LPD had significant improvement in creatinine, eGFR and blood glucose and nutritional status maintenance. On the other hand, patients with increased protein intake had eGFR reduction at the end of follow-up (only significant for DM patients).

The proposed mechanism by which the LPD can preserve renal function includes reduction of glomerular hypertension. A decrease in the glomerular capillary pressure reduces glomerular sclerosis, renal fibrosis, and proteinuria. In addition to this hemodynamic effect, the dietary protein intake might affect the accumulation of extracellular matrix protein directly or indirectly by abnormal tubular protein in the kidneys.^17^^,^^18^ Recently, an experimental study suggested that the renoprotective effect of the LPD could also be associated with the attenuation of the renal mammalian target of rapamycin (mTOR) pathway, a regulator of cellular protein synthesis, and cell growth.^19^

In fact, meta-analyses of studies of the effects of protein restriction on CKD progression in diabetic and non-diabetic patients showed beneficial results.^8^^,^^9^ However, a main concern regarding LPD is the development of protein energy wasting (PEW) with potential adverse consequences, such as an increased risk of death.^3^^,^^4^ In the present study, indicators of overall nutritional status (BMI) and visceral protein and also mortality (albumin) remained unaltered at the end of follow-up in the adherent groups (DM and non-DM).

Corrected implementation of LPD, which includes careful attention to amount of protein of high biological value, proper energy intake and to other nutrients, such as vitamins and micronutrients, is not able to induce PEW.^3^^,^^20^ On the other hand, excess dietary protein can have deleterious consequences, including accumulation of uremic toxins, as P-cresyl-sulphate, responsible for the well-known insulin resistance that occurs in many patients even in early stages of CKD.^20^ Increasing protein in the diet also increases the phosphates and salt intake as well as contributes to the generation of acid. These factors could lead to hyperparathyroidism, hypertension, loss of muscle mass and may even aggravate progression of CKD.^3–5^^,^^20^^,^^21^

Despite the effectiveness and safety of LPD, the adherence to the protein restriction is a key point on the management of nondialysis CKD patients.^12^^,^^13^^,^^22^ In the present study, demographic factors, such as age and gender, seem not to affect the low protein diet adherence. During the follow-up time, the dietitian improved the nutritional approach to increase the LPD adherence through some strategies: (a) adaptations in usual diet to incorporate the protein restriction to habitual meals and food habits (e.g., options for nocturnal snacking are provided for patients with prefer do not dining); (b) the use of replacement list of food to avoid diet monotony (e.g., one egg can be substituted by half steak portion); (c) use of portion sizes by measuring tools (with use of replicas of food and kitchen utensils) and photograph albums; (d) Analysis of the label of foods to patients learned where find the data about amount of protein per portion of processed foods; (e) stimulation to improve the meals with specific receipts of low protein content.^23^ In fact, to maintain the LPD over time, the diet prescribed has to be pleasant, varied and not too restrictive.^12^ Thus, the intensive counseling (up to 3 months apart) by a skilled dietitian, as performed in the present study, is highly recommended.^2^

The patients in groups G1, G2, and G4 had a significant improvement in the venous bicarbonate levels during the follow-up time. Curiously, non-diabetic LPD adherent patients (G3) presented adequate bicarbonate levels (>22 mM) and they not presented increasing in bicarbonate levels. This interesting feature could mean that even those patients considered non-adherent to LPD may have reduced their usual protein intake that contributed to increment in the bicarbonate values in G2 and G4 groups.

As well as protein intake, bicarbonate levels could be associated with CKD progression.^24^ Thus, multivariate analysis was performed to evaluate the association between some variables and the annual changes on eGFR. Age, gender, smoking status, BMI, lipid profile, and bicarbonate were not independent associated with changes in eGFR by year. Thus, protein intake could be the responsible for improvement on creatinine and eGFR in patients adherent to LPD (diabetics and non-diabetics). However, the absence of data about dietary protein intake (g/kg/day) is the main limitation of the present study because avoid more accurate evaluations regarding independent factors associated with CKD progression; or specific effects of protein intake and clinical outcomes, such as bicarbonate levels.

Moreover, the present study has others limitations. Firstly, the adherence to LPD was evaluated only by dietary methods because the 24-h urinary urea nitrogen excretion could not be determined due to logistical reasons. Although not considered the gold standard, the systematic use of dietary methods could be a strategy to assess the feasibility of dietary interventions because provides detailed intake data.^25^ Although dietary data were collected, just the categorizing in “adherent” and “nonadherent” patients is available. The availability of original data would provide important information about the real protein intake (g/kg/day) and its correlations to other variables, such as serum bicarbonate levels. Secondly, kidney function was estimated only through eGFR (based on serum creatinine) because cystatin C or others renal biomarkers are not available in clinical practice. Thirdly, clinical outcomes closely related to CKD progression (such as blood pressure control and proteinuria) could not be evaluated. Consequently, the effects of LPD in the outcomes above mentioned, as well as adjustments for all possible confounders could not be performed due to retrospective study design. In this specific matter, randomized study design had important ethical concerns because if LPD promotes potential benefits to the nondialysis CKD patients, should we randomize patients to a LPD or unrestricted diet?^14^

Even with all these limitations, the present study reports that low protein diet is nutritionally safe and it could have a positive effect on the improvement of creatinine and renal function in diabetics and non-diabetics patients. Additionally, nutritional intensive counseling is important to motivate the patients to adhere to dietary protein restriction. However, controlled studies are needed to evaluate the specific effects of the protein restriction on glomerular filtration rate and CKD progression.
